# Comparative Study of Modified Quantitative Buffy Coat and Two Rapid Tests in Comparison with Peripheral Blood Smear in Malaria Diagnosis in Mumbai, India

**DOI:** 10.1155/2014/194651

**Published:** 2014-03-27

**Authors:** Manali M. Kocharekar, Sougat S. Sarkar, Debjani Dasgupta

**Affiliations:** ^1^Institute of Science, 15 Madam Cama Road, Mumbai 400032, India; ^2^Pharmaceuticals Division, Novartis Healthcare Pvt Ltd., Sandoz House, Shivsagar Estate, Dr. Annie Besant Road, Worli, Mumbai 400018, India; ^3^Department of Biotechnology & Bioinformatics, Padmashree Dr. D.Y. Patil University, Sector 15, Plot No. 50, Central Business District, Belapur, Navi Mumbai 400614, India

## Abstract

In order to identify a quick and reliable technique for accurate diagnosis of malaria, study of the efficiency of the tests such as Parahit total (HRPII & aldolase Ag), Advantage mal card (parasite specific LDH), and modified QBC was done in comparison with conventional blood smear microscopy. One hundred patients infected with * P. vivax* and 101 infected with * P. falciparum* were included in this study. The sensitivity of Parahit total, Advantage mal card, and modified QBC for * P. falciparum* detection was 70.3, 95%, and 98%, and specificity was 98%, 98%, and 96%, respectively. The sensitivity of Parahit total, Advantage mal card, and modified QBC for * P. vivax* detection was 73%, 97.0%, and 98%, respectively, and specificity of all the tests was 98%. On day 15, in falciparum arm, Advantage mal card and Parahit total showed 8 (7.92%) and 59 (58.41%) false positives. On day 15, in vivax arm, Parahit total revealed 52% false positives. The study indicated that modified QBC could be only used where appropriate facilities are available. Advantage mal card was a better follow-up tool than Parahit total.

## 1. Introduction

The National Vector Borne Disease Control Programme (NVBDCP) of India reports about 2 million malaria parasite positive cases annually, of which about 50% are* Plasmodium falciparum* [1].

The gold standard for malaria detection is microscopic detection which is tedious and dependent on technical expertise. Therefore tests like Parahit total, Advantage mal card,and QBC have been introduced. In view of the seriousness of the malarial infection and paucity in current availability of diagnostic facilities across India, we have conducted a comparative study of the above tests with microscopy at the baseline and on eight and 15 days of followup.

## 2. Materials and Methods

The present study is a prospective, assessor blind, comparative study evaluating various techniques used for the diagnosis of malaria in patients suffering from uncomplicated symptomatic malaria due to* Plasmodium falciparum* or* P. vivax*, conducted after approval from Independent Ethics Committee.

### 2.1. Participants

Patients of either sex between 18 to 70 years of age with a history of fever suspected with malaria at the General or Malaria Outdoor Patient Department (OPD), Kasturba hospital for Infectious diseases, Mumbai, India, were screened after obtaining written informed consent from May 2010 to November 2011. Patients presenting with symptomatic, uncomplicated malaria confirmed by the presence of either or both of the following criteria were included:

- blood smears positive for* Plasmodium falciparum* or* Plasmodium vivax* asexual parasitemia and/or sexual parasites,
- fever or history of fever within the prior 24 hours.

Patients with severe clinical manifestations which require immediate referral were excluded from the study.

Informed consent was taken from all patients who participated in the study. The vital parameters such as temperature, pulse, and blood pressure were recorded at the start of the study (day one).

*Sample Collection*. Blood for study purpose was collected by finger prick method.

Two or three drops of blood were placed on a glass slide, five µL into pipette for Advantage mal card, five µL into applicator for Parahit total, and 55–65 *μ*L directly added into QBC tube and examined by blinded assessor. Patients were called for follow-up posttreatment on day eight and day 15. This was done to estimate the success of treatment indicated by the negative tests.

### 2.2. Test Methods

#### 2.2.1. Microscopic Diagnosis Using Stained Thin and Thick Peripheral Blood Smears (PBS)

Thick and thin film was prepared on the same slide stained by Giemsa method and examined under oil immersion lens by light microscopy.

Asexual- and sexual-stage parasite densities were determined from the thick films by counting the number of parasites separately against 200 leukocytes and were then expressed in microliters (*μ*L). If less than or equal tonine parasites were detected against 200 white blood cells, parasites were counted up to 500 white blood cells. Thick films were considered negative if no parasites were seen in at least 100 consecutive oil immersion fields.

#### 2.2.2. Quantitative Buffy Coat (QBC)

QBC (Becton Dickinson) employs microhematocrit centrifugation which is an effective means of detecting malarial parasites by direct examination. It employs a capillary tube which is internally coated with EDTA and acridine orange [2]. The use of the dye is based on the premise that infected red cells appear to be less dense than uninfected and is concentrated primarily within the zone at the interface—a small one to two mm region near the top of the RBC column. These parasites fluoresce as green and orange objects because of the uptake of dye.

#### 2.2.3. Modified QBC Technique

In modified QBC, QBC tube was filled with 55 *μ*L blood. Stopper and float was placed at either end of the tube and then centrifugation was done in locally available RM-12 C REMI microcentrifuge instead of parafuge at 12,000 RPM for five minutes. The centrifuged tube was placed in the paraviewer tube holder and examined under 60× objective of UV microscope manufactured by Labomed ltd. Parasite nucleus fluoresces bright green, while cytoplasm appears orange. The total examination time to exclude a negative was seven to ten minutes. Results for the above technique were reported in terms of the following: (1) presence or absence of parasite and (2) morphology.

#### 2.2.4. Evaluation of Malaria Rapid Diagnostic Tests (RDTs)

Both malaria RDTs were performed and interpreted according to the manufacturer's instructions.

*(1) Advantage Mal Card*. Batch number ACM15101, J Mitra & Co ltd, India, is an individually packaged test cassette, diagnosing Plasmodium infections by pLDH detection, distinguishing between* Plasmodium falciparum* and the other malaria species* Plasmodium vivax*,* Plasmodium malariae*, or* Plasmodium ovale*. It requires five *μ*L of whole blood to be collected with a pipette provided by the test kit. Test results need to be read after 20 minutes.

*(2) Parahit Total Test*. (Batch number 4000006623, SPAN Diagnostics Ltd, Surat, India.) This test was performed using commercially supplied test strips and reagents following the manufacturer's instructions. It is also an individually packaged strip diagnosing* Plasmodium falciparum* infections by HRP-II (histidine-rich protein-2) detection and Pan malarial antigen aldolase for other species. It requires five *μ*L of whole blood to be collected with a sample applicator provided by the test kit. Test results need to be read after 20 minutes.

#### 2.2.5. Visit Schedule

All patients were seen on day one and were asked to come for followup on days eight and 15. Each patients sample was subjected to all the tests on the days of followup.

The rapid tests were read by two independent blinded research assistants to minimize bias. The blood films were examined by a blinded experienced microscopist in the laboratory without reference to the results of Rapid tests and clinical history of patient. All negative slides that tested positive by the Rapid tests or all positive slides that tested negative by the Rapid tests were again examined by another expert microscopist blinded to the results of microscopy.

#### 2.2.6. Statistical Methods

Most of the analysis was done by Instat version 3.2 (GraphPad Software, California) and Epimax calculator (Clinical & Economic Software Solutions, New Jersey USA). Sensitivity was also calculated manually as TP/(TP + FN) × 100%, specificity as TN/(TN + FP) × 100%, the positive predictive value (PPV) as TP/(TP + FP) × 100%, the negative predictive value (NPV) as TN/(FN + TN) × 100%, false positive rate (FPR) as FP/(FP + TN) × 100%, accuracy (ACC) as (TP + TN)/(positive + negative) × 100%, and false discovery rate (FDR) as FP/(FP + TP) × 100%. Sensitivity and specificity were used to calculate the likelihood ratios for a positive test result [sensitivity/(1 – specificity)] and a negative test result [(1 – sensitivity)/specificity]. Odds ratio was also calculated. All the parameters of the tests were assessed with microscopic detection as the gold standard.

## 3. Results

### 3.1. Participants

**Figure 1:**
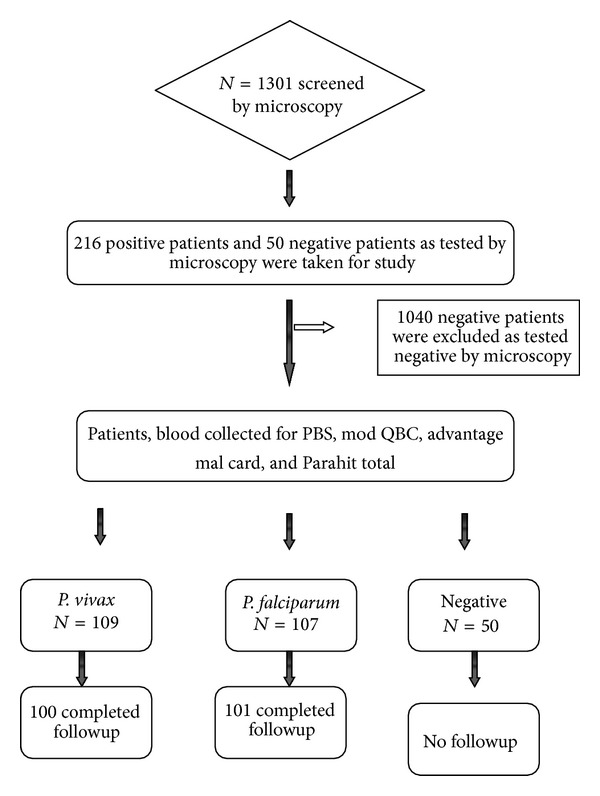
Flow chart of the study.

A total of 1301 patients who were suspected with malaria were screened for malarial parasites by thick and thin PBS (peripheral blood smear) from May 2010 to November 2011. After screening, 266 patients who fulfilled the inclusion criteria (informed consent, diagnosed to be positive, fever, or history of fever) were enrolled in the study; 216 patients were positive for malarial parasites; 50 were negative for malarial parasites. Out of 216 positive patients, 201 completed followup, 101 patients were infected with* Plasmodium falciparum,* and 100 were infected with* Plasmodium vivax* as shown in the Figure 1.

The age of the patients diagnosed with falciparum malaria ranged from 18 to 70 years, (31.94 ± 11.37) and that with vivax was 32.01 ± 12.21. The mean baseline, that is, on day one, asexual parasite density in falciparum group was 4436.16 ± 11996.87 (0–112000), sexual density was 335.2 ± 769.75 (0–6000 µL), mean asexual density in vivax group was 2728 ± 3157.15 (40–13040 µL), and sexual density was 3610 ± 4407.55 (80–19200 µL) (Table 1).

### 3.2. Test Results

Out of the 50 negative patients, one false positive was detected as compared with PBS microscopy. The sensitivity, specificity, PPV, NPV, and likelihood ratio positive and likelihood ratio negative of falciparum and vivax arm have been calculated using 2 × 2 table with Peripheral blood smear as gold standard (Tables 2, 3 and 4).

In the falciparum arm, Advantage mal card failed to detect five falciparum positives. On day 8, 9 patients with* P. falciparum* gametocytes were detected by Advantage mal card. Along with this, 15 false positives were also detected on day eight. On day 15, it detected 8 false positive which were negative by microscopy.

Parahit total failed to detect 30 falciparum positives. Parahit totaldetected 68 false positive on day 8 and 59 false positive on Day 15.

Modified QBC failed to pick up two positives on day one. On day eight, all nine patients with* P. falciparum* gametocytes were detected, whereas on day 15, all were negative for parasites.

Parahit total and Advantage mal card did not detect any positive at a parasite density less than 200 in the vivax arm, whereas QBC showed a sensitivity of 33%.

Parahit total showed a sensitivity of 75.25% at a parasite density more than 200 in the vivax arm, whereas Advantage mal card and QBC showed a sensitivity of 100%.

Parahit total showed a very low sensitivity of five percent at a parasite density less than 200 in falciparum arm, whereas Advantage mal card and QBC showed a sensitivity of 75% and 90%, respectively (Table 5).

Parahit total showed a sensitivity of 86.41% at a parasite density more than 200, whereas Advantage mal card and QBC showed a sensitivity of 100%.

In the vivax arm, out of the 100 complete follow-up patients, Advantage mal card failed to pick up 3 samples which were vivax positive by PBS microscopy. On day eight, four patients were false positive while on day 15 all the patients were negative for parasites.

Parahit total failed to pick up 27 samples which were vivax positive by microscopy. It detected 1 as mixed infection which was positive for* P. vivax* by microscopy.

On day eight, there were 67 false positives which were negative by microscopy and, on day 15, Parahit total detected 52 false positives.

Modified QBC arm on day one failed to pick up two samples which were detected positive for* P. vivax* by microscopy. It also detected one sample as false positive which was detected negative by microscopy.

The sensitivity and specificity of Parahit total was 73% and 98% in vivax arm and 70% and 98% in falciparum arm. Advantage mal card had a sensitivity and specificity of 97% and 98% in vivax arm and 95% and 98% in falciparum arm, respectively, when compared with peripheral blood smear. The sensitivity and specificity in vivax arm was 98% and in falciparum arm was found to be 98% and 96%, respectively, which makes it a good diagnostic test.

## 4. Discussion

The accurate diagnosis of malaria is important for the timely treatment of febrile patients with antimalarial drugs to reduce their mortality and morbidity and also to effectively manage nonfebrile illness. PBS microscopy is very tedious and time consuming. Various sensitive methods have been employed for the simple, reliable, and rapid diagnosis of malaria. The most promising of these were the rapid diagnostic tests and QBC [3] which were compared with Giemsa stained PBS microscopy for diagnosis of* P. vivax* and* P. falciparum* infections.

Modified QBC method failed to detect two PBS vivax positives. This may be due to the fact that the specific gravity of late trophozoites of* P. vivax* is similar to the leukocytic layer of the buffy coat causing the parasites to get obscured in the granulocytic layer following centrifugation. They also become difficult to identify as they get compacted and lose their amoeboid shape during centrifugation [4]. Modified QBC also failed to detect two PBS falciparum positives which may be due to low parasitaemia. First time users may also fail to identify parasites especially when the parasite concentration is low [5].

The sensitivity of QBC has been reported to be as high as 90%, by Gurung et al. [6], 96.22%, by Bhandari et al. [7], and 99.7%, by Benito et al. [8]. Similar sensitivity was found for modified QBC in our study. The sensitivity and specificity in vivax arm was 98% and in falciparum arm was found to be 98% and 96%, respectively, in the present study, which makes it a good diagnostic test.

Modified QBC detected one sample as false positive for* P. vivax* and two as false positives for* P. falciparum* which were negative by microscopy.

Results were also made available in just eight to 15 minutes which is a fraction of the time required for thick film methods. The resources for training were also reduced as trainees with 3–5 days of training could produce results comparable to an experienced microscopist. Modified QBC holds promise as a good alternative to Giemsa stained PBS due to its speed, sensitivity, and specificity as routine QBC especially where the patient load is extremely high.

Our modification of the routine QBC method reduces the cost per test by 48% in a small laboratory setup [9].

Of the two antigen tests evaluated, Advantage mal card showed a sensitivity and specificity of 97% and 98% in vivax arm and 95% and 98% in falciparum arm, respectively, when compared with peripheral blood smear. Sensitivity of Advantage mal card test was almost similar when compared to QBC. The limitation of antigen test is that it cannot distinguish between active infection and recently treated infection which still remains an important advantage of microscopy and QBC. In addition, RDT cannot detect the severity of disease and is only useful in diagnosis of malaria. In addition parasite count also cannot be done using RDT which is especially required for* P. falciparum* infection.

The sensitivity and specificity of Parahit total were 73% and 98% in vivax arm and 70% and 98% in falciparum arm. The sensitivity of Advantage mal card was far better than Parahit total for detecting malarial infections. The low sensitivity for* P. falciparum* by Parahit total may be because there were 17 patients with only gametocytes which were not detected by Parahit total. Since the antigen (Pf HRP-II) is present only in asexual erythrocytic stages and to a certain extent in early gametocytes, the test does not detect patients in the prepatent stage of malaria or those who have only mature gametocytes in their blood [10].

The major benefit of Advantage mal card (pLDH Ag) over Parahit total (HRP-II Ag) is that there were less false positives in the follow-up studies.

The high false positivity in falciparum arm in Parahit total may be explained by the fact that the body slowly eliminates HRP-II after parasite clearance. HRP-II has been shown to persist and is detectable after clinical symptoms of malaria have disappeared and the parasites have apparently been cleared from the host [11]. Humar et al. detected circulating HRP-II antigen in 68% of treated patients on day seven and in 27% on day twenty-eight. The persistence of HRP-II is still unclear [12].

As suggested by Gerstl et al. [13] in endemic areas, it is important to have short parasite antigen clearance time after the parasites are cleared so that the health care person can interpret Rapid test results. The false positive results from previously treated infections are thereby eliminated.

Ashley et al. [14] have suggested that the sensitivity of OPTIMAL which detects pLDH antigen in vivax monoinfections was 92.2%. The sensitivity of CareStart (detects pLDH antigen) was 93.5% for detection of falciparum infections but performed poorly for the detection of nonfalciparum infections with a sensitivity of only 78.5% (95% CI = 73–83.1) which limits its utility in areas with a high prevalence of nonfalciparum infections [14]. In our study the sensitivity of* P. vivax* detection was more than* P. falciparum* thus making it the only Rapid test with such a high sensitivity for* P. vivax*.

In the present study, Advantage mal card showed sensitivity of 97% for* P. vivax*, thus making it a good diagnostic tool in areas where the predominant species is* P. vivax*, as in Mumbai, India. Thus Advantage mal card clearly has advantage over Parahit total and also on other malaria pLDH detection tests especially for nonfalciparum infections. Few RDTs have reported over 90% sensitivity for* P. vivax*. However this study shows that Advantage mal card has a very high sensitivity and specificity for both* P. vivax* and* P. falciparum* which may be attributed to improved diagnostic technology since all our tests were carried out in hospital settings following proper storage conditions and manufacturer's instructions. The results of all RDTs are comparable.

## 5. Conclusion

There is need to employ more sensitive tests which apart from being rapid will also be able to detect low levels of parasitaemia. The alternatives to Giemsa stained microscopy are PCR, QBC, and RDTs. PCR is unsuitable for routine use in the field or clinical laboratory as it is a research tool. The QBC method was found to be more rapid than peripheral blood film examination as centrifugation causes the malarial parasites to concentrate to a small compact zone below the buffy coat layer. This increases the speed and ease of interpretation especially in the case of low parasitaemia. Background and morphological appearances are not improved by modified QBC. Background and morphological appearances of routine QBC are almost similar to modified QBC. Our modification of the routine QBC technique renders it cheaper without compromising the sensitivity and specificity of the technique. It is noninferior and as sensitive as routine QBC. It is also more cost effective than routine QBC which is a requirement for diagnostic laboratories in India. Therefore modified QBC could be the method of choice for laboratory setting in India as an alternative to conventional microscopy. However Giemsa stained microscopy is the gold standard.

There is increased investment in antimalarial drug development but it should be accompanied by parallel investments in improving diagnostic tools. Considering the advantages and disadvantages of the diagnostic methods, it can be concluded from this investigation that modified QBC can replace microscopy in setups where appropriate facilities are available. However in situations where adequate laboratory backup is not available, simpler and user friendly techniques like Advantage mal card can be employed which has high sensitivity and specificity. The result of the present study would also be helpful in making policy decisions for national and international malaria eradication programmes.

## Figures and Tables

**Table 1:** Baseline characteristics of included patients.

Parameters	Falciparum arm (*n* = 101)	Vivax arm (*n* = 100)	Negative arm
Age (mean ± SD) in years	31.94 ± 11.37	32.01 ± 12.21	28.72 ± 10.36
Sex: male : female	96 : 5	97 : 3	39 : 11
Asexual parasite density (mean ± SD)	4436.16 ± 11996.87	2728 ± 3157.15	0
Gametocyte density (mean ± SD)	335.2 ± 769.75	3610 ± 4407.55	0
Duration of fever (mean ± SD) in days	3.45 ± 1.55	2.86 ± 0.94	1.9 ± 0.64

**Table:** (a)

Giemsa smear	Advantage mal card		Parahit total	
	Positive	Negative	Positive	Negative
Vivax				
Positive-100	TP 97	FN 3	73 TP	27 FN
Negative-50	1 FP	TN 49	1 FP	49 TN
Total-150	** 98**	**52**	**74**	**76**
Falciparum				
Positive-101	96 TP	FN 5	71 TP	30 FN
Negative-50	1 FP	TN 49	1 FP	49 TN
Total-151	** 97**	**54**	**72**	**79**

**Table:** (b)

Smear	Modified QBC	
	Positive	Negative
Vivax		
Positive-100	98 TP	2 FN
Negative-50	1 FP	49 TN
Total-150	** 99**	**51**
Falciparum	Positive	Negative
Positive-101	99 TP	2 FN
Negative-50	2 FP	48 TN
Total-151	** 101**	**50**

TP: true positive, TN: true negative, FN: false negative, and FP: false positive.

**Table 3:** Patients infected with *P.  falciparum* *N* = 101.

	Modified QBC	PLDH	Parahit total
Sensitivity	98 (94.4–99.5)^ab^	95.0 (91.3–96.0)^∗a^	70.3 (65.9– 71.2)^∗b^
Specificity	96 (88.7–99.0)^cd^	98.0 (95.1–99.9)^c#^	98 (89.2–99.9)^#d^
Positive predictive value	98 (94.4–99.5)	99.0 (95.1–99.9)	98.6 (92.5–99.9)
Negative predictive value	96 (88.7–99.0)	90.7 (83.7–92.5)	62.0 (56.4–63.2)
Likelihood ratio +	24.5	47.52	35.14
Likelihood ratio −	0.02	0.050	0.30
Odds ratio	1176 (131.09–18730.21)	940.80 (99.17–22239.18)	115.96 (15.96–2389.96)
Relative risk	24.50 (8.35–97.02)	10.68 (5.84–13.31)	2.59 (2.12–2.71)
Kappa	0.94 (0.831–0.985)	0.912 (0.801–0.940)	0.59 (0.48–0.62)
Overall accuracy	0.9733	96.0	0.79
False positive rate	0.04	0.02	0.02
False negative rate	0.02	0.049	0.29

*Statistical significant *P* = 0.0001 highly significant and ^#^not statistical significant *P* = 1.

^a^Not statistically significant *P* = 1.0000, ^b^extremely statistically significant. *P* value is less than 0.0001, ^c^not statistically significant *P* value = 1.0000. ^d^Not statistically significant *P* value = 1.0000.

**Table 4:** Patients infected with *P.  vivax* *N* = 100.

	Modified QBC	PLDH	Parahit total
Sensitivity	98 (94.5–98.9)^ab^	97.0 (93.4–97.9)^∗a^	73% (68.6–73.9%)^∗b^
Specificity	98 (91.1–99.9)^cd^	98.0 (90.8–99.9)^#cd^	98% (89.2–99.9%)^#d^
Positive predictive value	99 (95.5–99.9)	99.0 (95.3 –99.9)	98.6 (92.7–99.9)
Negative predictive value	96.1 (89.3–97.9)	94.2 (87.3–96.1)	64.5 (58.7–65.7)
Likelihood ratio +	49	48.5	36.5
Likelihood ratio −	0.02	0.030	0.27
Odds ratio	2401 (177.42–87320.96)	1584.33 (140.25–44349.3)	132.48 (18.11–2739.27)
Relative risk	25.24 (8.93–48.37)	17.15 (7.52–25.30)	2.77 (2.24–2.91)
Kappa	0.95 (0.85–0.98)	0.941 (0.834–0.969)	0.628 (0.51–0.65)
Overall accuracy	98	97.33	81.33
False positive rate	0.02	0.02	0.02
False negative rate	0.02	0.03	0.27

*Statistically significant *P* = 0.0001 highly significant.

^#^Not statistically significant *P* = 0.062, ^a^not statistical significance *P* = 1.0000.

^c^Not statistical significant *P* = 1.0000, ^d^not statistical significance *P* = 1.0000.

^b^ *P* value is less than 0.0001. It is extremely statistically significant.

**Table 5:** Sensitivity of RDTs and QBC at parasite density above and below 200/*μ*L.

Tests	Microscopy result			
	PF < 200/*μ*L *N* = 20	PF ≥ 200/*μ*L *N* = 81	PV < 200/*μ*L *N* = 3	PV ≥ 200/*μ*L *N* = 97
Parahit total	1 (5%)	70 (86.41 %)	0	73 (75.25%)
Advantage mal card	15 (75%)	81 (100%)	0	97 (100%)
QBC	18 (90%)	81 (100%)	1 (33.33%)	97 (100%)

## Key Messages

More investments are required to improve tests for malaria diagnosis. This work indicates that modified QBC is a cost effective, accurate test which can be used in laboratories with appropriate facilities. Advantage mal card is a better follow-up test but which can be used even without a laboratory setup.
